# Exploring the perception of barriers to a dual career by student-athletes with/out disabilities

**DOI:** 10.1371/journal.pone.0286152

**Published:** 2023-05-19

**Authors:** Raquel Vaquero-Cristóbal, María J. Maciá-Andreu, Álvaro Díaz-Aroca, Lourdes Meroño, Juan Alfonso García-Roca, Lucía Abenza-Cano, Francisco J. Cánovas-Álvarez, Antonio Sánchez-Pato, Alejandro Leiva-Arcas

**Affiliations:** 1 Facultad de Deporte, UCAM Universidad Católica de Murcia, Murcia, Spain; 2 Facultad de Ciencias de la Salud, Universidad Internacional de La Rioja, Madrid, Spain; University of Extremadura: Universidad de Extremadura, SPAIN

## Abstract

In recent years, there has been an increase in knowledge about the barriers experienced by people with disabilities in the education system or sports. However, no studies have analyzed the barriers for those who try to succeed in both disciplines (dual career). The purpose of this study was to examine the barriers faced by student-athletes with/out disability to a dual career combining studies and sport. Two groups were involved in the study (n = 162): student-athletes with disabilities (n = 79) and student-athletes without disabilities (n = 83). Data collected included: (a) socio-demographic aspects; and (b) barriers towards achieving a good balance between sport and academics during the dual career, through the "Perceptions of dual career student-athletes" (ESTPORT) questionnaire. The results showed that student-athletes with disabilities were more likely to perceive in a greater extent the barriers, *the university is far from my home* (p = 0.007) and *the university is far from my training site* (p = 0.006), *I find myself unable to balance study and training time* (p = 0.030), *I have to take care of my family* (p<0.001), and *my current job does not allow me to study enough* (p<0.001). The MANOVA analysis showed that the factors gender, competitive level, and employment status had an influence on the perception of some barriers between groups. In conclusion, student-athletes with disabilities perceived barriers more strongly than those without disabilities, and measures are needed to ensure their inclusion in the education system.

## Introduction

The sports career of elite athletes ends prematurely as compared to other professional careers, as the elite sport career entails five to ten years dedicated to sport, reaching the mastery of their athletic level before the age of 30, when the discontinuation period begins, so it is common to find that in many cases, it is necessary to opt for another type of work activity after retiring from sports [1–3]. To make this possible, the dual career of student-athletes, understood as the process of combining a sports career with an academic career or work, is the best option for the best transition after retirement from sports [4], as combining studies with a sporting career allows the athlete to better prepare for future employment [5]. However, in the case of student-athletes with disabilities, in addition to trying to succeed by combining their sports and academic careers, which is already very difficult, they must face an added disadvantage, as they are often affected by the system’s lack of inclusion of them [6, 7], which is reflected in the barriers they face in achieving success in both academic and sporting fields.

However, most of previous studies have focused on non-disabled student-athletes, with little literature addressing barriers in athletes with disabilities. In addition, classically the structure of barriers used in the analysis of disabled student-athletes has been based on the findings of non-disabled student-athletes [8, 9]. In this line, different studies have classified the main barriers that student-athletes without disabilities face when trying to achieve a successful dual career combining studies and sport into two main categories: those external to the athlete, and those that are internal [10]. With regard to external barriers, some of the barriers that student-athletes must face are the lack of flexible structures for combining academic and sports progress [11, 12], the lack of tools for a good planning of their schedule [13], the absence of support staff from the university to advise and guide the student-athlete throughout the process [14], or funding problems during the dual career combining studies and sport [15], as a large number of them receive some kind of institutional scholarship conditioned on obtaining good results both athletically and academically, and scholarships are usually partial [16, 17]. In terms of internal barriers, stress and its management are the main aspect mentioned by student-athletes [18]. Park et al. [19] identified the main critical factors that could increase the stress of student-athletes, including the occurrence of injuries, health problems, control over their life, self-perception, relationship with their environment, changes in their life, or identity conflicts among others. Consequently, the occurrence of acute stress episodes resulting from periods of competition or exams may lead to the abandonment of dual careers [20] mainly because of the inability to manage the time dedicated to studying and training due to external time constraints [21]. This is because most individuals pursuing dual careers consider themselves to be athletes rather than students [22]. Research such as that by Cosh and Tully [13] found that in high-stress situations, there is a tendency to prioritize the sport dimension over the academic one, with the resulting negative impact on educational performance.

Given the lack of a sufficient number of studies that analyze barriers in both educational and sporting environments of student-athletes with disabilities, it has been proposed to use the model found in athletes without disabilities to cover this topic [8, 9]. However, there are no known studies that have analyzed the barriers of disabled student-athletes to success in their dual careers despite this being a necessity to be able to intervene from the educational and sporting spheres in order to facilitate the compatibility of both tasks [9]. In this sense, previous studies have presented a partial view, where barriers to success in education for people with disabilities, regardless of their status as athletes or not, have been analyzed [6, 9, 23–25]; or the barriers to success for athletes with disabilities in sport have been analyzed, without taking into consideration their academic career [26–32].

More specifically, previous studies have highlighted the barriers of people with disabilities in achieving academic success due to the limiting barriers they encounter in external factors such as society itself [6], university opportunities for students with disabilities [23], physical barriers in education facilities [23], lack of scholarships and financial support [9, 33], lack of individualization of education and procedures from professors and academic staff [24, 25, 34]. Regarding the studies that have analysed the difficulties for the success of sports careers exclusively among disabled athletes, regardless of their student status, they have pointed out as main external barriers the lack of opportunities to participate in sports, train, or compete in adapted clubs [26], the absence of policies for the promotion of disabled sport [27], the difficulties in traveling and the distances between activities [28, 30, 32], or lack of support from family, friends or the community as a consequence of their dependence extending into adulthood [28, 29, 31].

However, the absence of studies that have analyzed barriers to the success of dual careers in student-athletes with disabilities means that there are no data on some internal barriers that have showed a lot importance in the dual career of non-disabled athletes such as the interference of sporting performance with their studies and vice versa, as well as the need to prioritize one over the other o the distribution of time between study and training [13, 28]; or the disregard of their rights under existing national and international policies for the protection of dual careers [33]. This can also be made more difficult by the fact that some student-athletes need to work in order to earn an income due to the lack of professionalism in the sports field, which causes a new internal barrier to appear in reference to the need to distribute time between these three demanding activities (studies, sport and work), having to sacrifice one in order to achieve success in the others, which often leads to failure in the academic field [9, 31]. However, there has been no analysis of how this might affect disabled students-athletes-workers.

For all of the above reasons, it is necessary to address this gap in science, which is the absence of studies on the barriers experienced by disabled student-athletes, using non-disabled athletes as a theoretical framework on which to base it and with which to compare the incidence of these barriers in this population [8, 9], also taking into account socio-demographic and sporting variables that could modulate this perception of barriers, such as gender, level of sport or employment status, among others [35]. This research approach would be interesting to better understand the main challenges of a dual career faced by athletes with disabilities, as compared to non-disabled athletes, and to set a starting point for making the necessary adaptations and including them in the education and sports system with full guarantees of success. Therefore, the objectives of this research were: 1) to determine the differences between disabled and non-disabled student-athletes in the perception of barriers in the pursuit of success in the dual career depending on socio-demographic and sport characteristics, in order to identify the aspects that could be hindering their inclusion with guarantees into the educational system; and 2) to analyse whether socio-demographic and sporting variables such as gender, sporting level or employment status of student-athletes could influence the perception of barriers of disabled and non-disabled student-athletes. The hypothesis of this research was that student-athletes with disabilities will perceive barriers to a larger extent and have more difficulties in the pursuit of dual career success, and that some socio-demographic and sporting variables could modulate the barriers encountered.

## Materials and methods

### Design

The study used a descriptive and cross-sectional design, employing a non-probability convenience sampling approach. Adherence to the STROBE statement [36] was observed throughout the research design and manuscript development. Before data collection, study participants provided written consent and received information regarding the research objectives and the confidentiality of data obtained. The institutional ethics committee evaluated and approved the data collection protocol (code: CE012101) in compliance with the guidelines set forth by the World Medical Association and the Declaration of Helsinki.

### Participants

The sample size was calculated using Rstudio 3.15.0 software (Rstudio Inc., USA). The significance value was set at p = 0.05. The standard deviation (SD) was established considering the barriers from previous studies (SD = 0.75) [35]. With an estimated error (d) of 0.16, the required sample size for a 99% confidence interval (CI) was 79 subjects.

**Fig 1:**
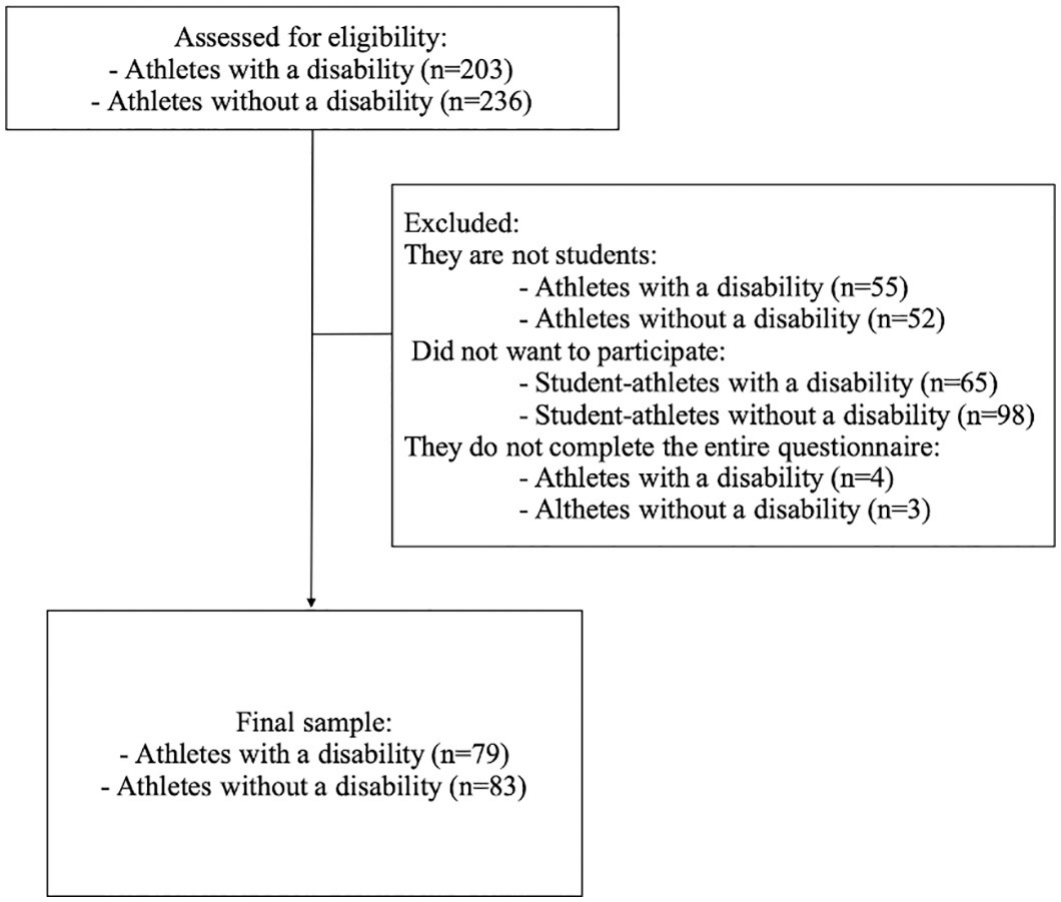
Flow diagram for the sample.

The inclusion criteria were: a) being an athlete; b) currently enrolled in post-compulsory education; c) being in the database of athletes of the Spanish Olympic Committee and the Spanish Paralympic Committee, including those that are currently competing in this competition or may be likely to do so in the future for having achieved great success in national or international championships; and d) training and competing normally. The exclusion criterion was not having completed the entire questionnaire. The flow diagram for the sample is shown in Fig 1.

The final sample consisted of 79 student-athletes with disabilities and 83 student-athletes without disabilities. Sample characteristics are shown in Table 1. On the one hand, regarding the characteristics of athletes with disabilities, they had a mean age of 24.34±5.80, were mostly male (65.8%), predominantly competing in individual sport (87.3%), and training/competing an average of 16.13±9.06 hours per week. On the other hand, non-disabled athletes had a mean age of 24.07±5.59, were mostly female (57.8%), predominantly competing in individual sport (59.0%), and training/competing an average of 18.30±6.49 hours per week. Significant differences were found between both groups in gender (p = 0.003); sport practiced (p<0.001); and highest level of competition (p<0.001).

**Table 1:** Differences between athletes with and without disabilities in socio-demographic, education, sports and employment situations.

		Athletes with disabilities (n = 79)	Athletes without disabilities (n = 83)	Adj. Res. with/without disabilities	Group differences (t, df, p; χ^2^, df, p)	Cohen’s d / Cramer’s V
Socio-demographic	Age	24.34±5.80	24.07±5.59	-	t = 0.30; df = 160; p = 0.764	-
	Gender	Male: 52(65.8%) Female: 27(34.2%)	Male: 35(42.2%) Female: 48(57.8%)	3.0 / -3.0 -3.0 / 3.0	χ^2^ = 9.11; df = 1; p = 0.003**	V = 0.24
Education	What do you study?	Vocational Education: 29(36.7%) Bachelor’s Degree: 38(48.1%) Master’s degree/Ph.D.: 12(15.2%)	Vocational Education: 6(7.2%) Bachelor’s Degree: 68(81.9%) Master’s degree/Ph.D.: 9(10.8%)	4.6 / -4.6 -4.5 / 4.5 0.8 / -0.8	χ^2^ = 26.52; df = 3; p<0.001***	V = 0.38
Sport career	Sport practiced	Individual: 69(87.3%) Collective: 10(12.7%)	Individual: 49(59.0%) Collective: 34 (41.0%)	4.0 / -4.0 -4.0 / 4.0	χ^2^ = 16.39; df = 1; p<0.001***	V = 0.32
	Highest level of competition participated in	PG: 23(29.1%) World Championships: 25(31.6%) European Championships: 1(1.3%) National Championships: 30 (38.0%)	OG: 17(20.5%) World Championships: 37 (44.6%) European Championships: 16(19.3%) National Championships: 13 (15.7%)	1.3 / -1.3 -1.7 / 1.7 3.7 / -3.7 3.2 / -3.2	χ^2^ = 23.09; df = 3; p<0.001***;	V = 0.38
Work	Do you work?	Yes: 19(24.1%) No: 60(75.9%)	Yes 27(32.5%) No: 56(67.5%)	-1.2 / 1.2 1.2 / -1.2	χ^2^ = 1.43; df = 1; p = 0.232	-
Distribution of time	How many hours do you spend per week studying/going to class?	25.16±16.55	20.53±14.36	-	t = 1.91; df = 160; p = 0.058	-
	How many hours do you train / compete per week?	16.14±9.06	18.30±6.49	-	t = -1.75; df = 160; p = 0.082	-

OG: Olympics Games; PG: Paralympic Games;

*p<0.05;

***p<0.001;

-: no significant differences.

### Measures

The "Perceptions of dual career student-athletes" (ESTPORT) questionnaire [37] was used to carry out the data collection. This is a validated questionnaire [37] used in previous research in Spanish context [16, 35, 38], which allows measuring the perception and barriers of student-athletes regarding their dual career. The internal consistency of the questionnaire is high, as Cronbach’s alpha coefficients are above 0.70 [37], with this being the lowest limit accepted as reliable [39]. This questionnaire is made up of 84 items with different types of response (Likert scale, multiple choice, and short answer), predominantly Likert scale. To obtain information about sociodemographic and contextual variables such as gender, age, sport career, education, or work, the following questions were asked (the number of the question corresponds to the original version of the questionnaire): 1 (Gender? Answers: Male; Female), 2 (Age?), 5 (Which sport do you practice? Answers: Individual; Collective), 6 (What is the highest level of competition at which you can compete? Answers: Olympic Games/Paralympic Games, World Championships, European Championships, National Championships, and Local Championships), 9 (What do you study? Answers: Vocational Education, Bachelor’s Degree, Master’s Degree/Ph.D.), 14 (Do you work? Answers: Yes; No. If Yes: How many hours a week?), 41 (How many hours do you spend per week studying?) and 61 (How many hours do you train per week?) were included. Furthermore, to know the difficulty of combining sports and academic life, question 20 (How easy/difficult is it for you to balance your sporting life with your academic life? Answers: 1: Very easy; 2: Easy; 3: Neither easy nor difficult; 4: Difficult; and 5: Very difficult) was also included. Finally, to discover the barriers, the scores obtained in items 26 to 37 of the questionnaire (26. The university is far from my home; 27. The university is far from my training site; 28. I find myself unable to balance study and training time; 29. My current job does not allow me to study enough; 30. I have to take care of my family; 31. I am usually tired; 32. I lose the rhythm of the course; 33. I lose touch with my classmates; 34. The cost of education is high; 35. I do not have enough university support; 36. Student schedules are not flexible; and 37. Training schedules are not flexible, following by is a barrier towards achieving a good balance between my sporting life and my studies. Answers: 1. Strongly disagree; 2. Disagree; 3. Neither disagree nor agree; 4. Agree; 5. Strongly agree). Cronbach’s alpha coefficient of the scale corresponding to the barriers with the sample used in this research was 0.82, understood as a high reliability [39]. The questions-and-answers structure was like that presented in previous studies [35, 38].

### Procedure

For the distribution of the questionnaire, and within the framework of the collaboration agreement established between the Catholic University of Murcia and the Spanish Olympic Committee, the Spanish Olympic Committee and the Spanish Paralympic Committee sent the questionnaire by email to all the athletes included in their databases, specifying in the email that the questionnaire should only be completed by those who were currently enrolled in vocational education (pre-university education), a Bachelor’s Degree, or a Master’s Degree/PhD. The questionnaires were sent out from 13 to 17 September 2021 and athletes had until 29 October 2021 to reply.

Initially, participants were required to review and sign the informed consent form outlining the research objectives and procedures. At this point, athletes were reminded that they should only complete this questionnaire if they were students, and that they had to confirm their status to continue with the questionnaire. Afterward, they anonymously and independently completed the questionnaire without any academic or competitive pressure, and in the absence of their coaches or professors. The questionnaire’s purpose was not elaborated upon beyond the information provided within the questionnaire itself. The survey was made available via the Google Forms^®^ platform and was completed by the participants in approximately 15 minutes. All data were collected anonymously.

The datasets generated for this study are available from the Zenodo database (DOI: 10.5281/zenodo.7863229).

### Statistical analysis

At the outset, the normality, homogeneity, and sphericity of the data were evaluated using the Kolmogorov-Smirnov, Levene’s, and Mauchly tests, respectively. Since all the variables analyzed showed a normal distribution, parametric tests were used. Descriptive statistics, such as mean values and standard deviation, were utilized to analyze quantitative variables, whereas qualitative variables were assessed using frequencies and percentages. An independent sample Student’s t-test was employed to identify any differences in the barriers between athletes with and without disabilities. Cohen’s d was calculated to find the effect size (ES) in these cases, with the following values utilized: small when d<0.2; moderate when d<0.8; and large when d>0.8 [40]. A MANOVA analysis was conducted to examine the influence of gender, level of sport, or employment status in the differences found between athletes with disabilities and those without disabilities regarding barriers. Following the initial analysis, a Bonferroni post-hoc analysis was conducted. The effect size (ES) was determined using partial eta squared (η2) and classified as small (ES ≥ 0.10), moderate (ES ≥ 0.30), large (ES ≥ 1.2), or very large (ES ≥ 2.0), with a p-value error of less than 0.05 [41]. A chi-square analysis (χ^2^) made it possible to establish the differences in the questions with a non-numerical, ordinal or nominal qualitative scale, between athletes with and without disability. Cramer’s V was used for the post hoc comparison of the 2x2 tables, and the contingency coefficient was used in the 2xn tables, to obtain the statistical value. The maximum expected value was 0.707; r<0.3 indicated a low association; r<0.5 indicated a moderate association; and r>0.5 indicated a high association [42]. A p<0.05 value was set to determine statistical significance. The statistical analysis was performed using the SPSS statistical package (v.25.0; SPSS Inc., IL).

## Results

The overall perception of the ease/difficulty of achieving dual career balance, as well as the main barriers encountered in achieving this balance, can be found in Table 2. It should be noted that the scores for the barriers *the university is far from my home* and *the university is far from my place of training* in the group of athletes with disabilities were significantly higher than in the group of athletes without disabilities (p = 0.004 and p = 0.002, respectively), as well as the barrier *I find myself unable to combine study time with training time* (p = 0.018), *my current job does not allow me to study enough* (p<0.001), and *I have to take care of my family* (p = 0.001). No differences were found for the remaining barriers.

**Table 2:** Comparison between disabled and non-disabled athletes in the overall perception of balance in the dual career and barriers to achieving that balance.

		Athletes with disabilities (n = 79)	Athletes without disabilities (n = 83)	Group differences (t, df, p)	Cohen’s d
Overall perception of balance (from 1 to 5)	How easy/difficult is it for you to balance your sporting life with your academic life?	3.32±0.92	3.57±0.98	t = -1.67; df = 160; p = 0.098	-
Barrier towards achieving a good balance between sporting life and studies (from 1 to 5)	The university is far from my home	3.05±1.49	2.40±1.57	t = 2.71; df = 160; p = 0.007**	0.42
	The university is far from my training site	3.16±1.51	2.48±1.57	t = 2.81; df = 160; p = 0.006**	0.44
	I find myself unable to balance study and training time	2.48±1.31	2.07±1.06	t = 2.19; df = 160; p = 0.030*	0.37
	My current job does not allow me to study enough	2.58±1.34	1.81±1.16	t = 3.94; df = 160; p = 0.000***	0.34
	I have to take care of my family	1.94±1.24	1.31±0.78	t = 3.84; df = 160; p = 0.000***	0.61
	I am usually tired	2.90±1.34	3.06±1.29	t = -0.78; df = 160; p = 0.437	-
	I lose the rhythm of the course	2.89±1.37	2.75±1.33	t = 0.655; df = 160; p = 0.513	-
	I lose touch with my classmates	2.81±1.43	2.80±1.54	t = 0.06; df = 160; p = 0.949	-
	The cost of education is high	2.51±1.43	2.90±1.63	t = -1.65; df = 160; p = 0.102	-
	I do not have enough university support	3.13±1.32	2.94±1.46	t = 0.85; df = 160; p = 0.396	-
	Student schedules are not flexible	3.24±1.32	2.87±1.55	t = 1.65; df = 160; p = 0.102	-
	Training schedules are not flexible	2.71±1.39	3.06±1.46	t = -1.57; df = 160; p = 0.119	-

*p<0.05;

**p<0.01;

***p<0.001;

-: no significant differences.

About the results of the MANOVA analysis, it was found that gender*group only showed a significant effect in *I do not have enough university support* (F = 4.84; p = 0.033; η^2^ = 0.10). The subsequent Bonferroni adjustment showed that females with disabilities perceived that they had significantly less support from the university than females without disabilities (3.63±1.41 Vs 2.53±1.23; p = 0.049; ICC95% = 0.01; 2.18).

About maximum level of competition*group only showed a significant effect in *I have to take care of my family* (F = 4.42; p = 0.019; η^2^ = 0.18) and *student schedules are not flexible* (F = 4.41; p = 0.019; η^2^ = 0.18). The subsequent Bonferroni adjustment found that disabled athletes who had competed in National Championships at maximum level showed higher scores on the barrier *I have to take care of my family* than non-disabled athletes at the same competitive level (2.50±1.29 Vs 1.14±0.38; p = 0.009; ICC95% = 0.35;2.36). On the other hand, it was in the group of OG/PG athletes that differences were found in the scores obtained in the barrier *student schedules are not flexible*, with the athletes with disabilities showing significantly higher scores (3.88±1.35 Vs 1.50±1.00; p = 0.008; ICC95% = 0.67; 4.08).

About employment status*group, significant differences in hours spend working and in the barrier *the university is far from my training site* (F = 6.24; p = 0.016; η^2^ = 0.12). The subsequent Bonferroni adjustment showed that student-athlete-workers with disabilities worked significantly more hours than those without disabilities (30.37±13.09 Vs 20.33±13.78; p = 0.017; ICC95% = 1.88; 18.19) and who had higher scores on the barrier *the university is far from my training site* (3.68±1.34 Vs 2.52±1.69; p = 0.016; ICC95% = 0.22; 2.11).

## Discussion

The main objective of this study was to determine the differences between student-athletes with and without disabilities regarding the perception of barriers in the pursuit of success in the dual career, in order to identify the aspects that could be hindering their inclusion in the education system with guarantees. The hypothesis was that student-athletes with disabilities will perceive the barriers to a greater extent and have more difficulties in the pursuit of dual career success. It was found that there were both internal and external barriers in which athletes with disabilities had higher scores than athletes without disabilities. The secondary objective of the present research was to analyse whether socio-demographic and sporting variables such as gender, sporting level or employment status of student-athletes could influence the perception of barriers of disabled and non-disabled student-athletes. The hypothesis was that some socio-demographic and sporting variables could modulate the barriers encountered. It was found that females with disability, disabled athletes who had competed in National Championships or PG at maximum level, and student-athletes-workers with disability showed higher scores in barriers than non-disabled.

More specifically, it was found that in the group of athletes with disabilities, the scores for some external barriers as *university is far from my home* and *university is far from my training site* were significantly higher than in the group of athletes without disabilities. In this regard, some of the barriers faced by this group were those of a geographical and logistical nature for the athlete, with the increased complexity and costs of transport, especially if it must be adapted [28]. For athletes with disabilities, issues related to the difficulty of getting to the place of training or study, the lack of a car park for people with disabilities, and the distances between journeys, among others, were stress factors [32], especially for those who moved in wheelchairs or had significant mobility limitations [30].

Regarding internal barriers, student-athletes with disabilities showed significantly higher values than non-disabled student-athletes in the item *I find myself unable to balance study and training time*. This circumstance affects student-athletes in general, although it is more notable as one of the barriers of personal nature, and the environment of the athlete with a disability, who has difficulties in reconciling sports practice with study or work, especially when dealing with a high performance or high-level sport [28]. The differences found between non-disabled and disabled athletes in this parameter could be due to athletes with disabilities experiencing greater difficulties in achieving success in both sports and university [6], to which we can added the fact that they are usually more protected by the family environment [43], which in this context could be a disadvantage, as they do not feel capable of facing this task.

Another interesting finding of this study was that student-athletes with disabilities scored the internal barrier *I have to take care of my family* significantly higher than non-disabled athletes. For athletes with disabilities, the support provided by family, friends or the community is essential when practicing sports, especially at the beginning, and for some more severe disabilities, throughout their sports life [29]. At this point, it is important to highlight parents, who play a vital role and support, in some cases, the main investments of the sports career [44], with a greater presence and involvement of parents in sport activities when their child has a disability [45]. On the other hand, the attitude of the family is also one of the personal and environmental barriers for athletes with disabilities [28]. In this regard, previous research has found that athletes with disabilities experience organizational stress related to their parents in adulthood, whereas these stressors stop at around 18 years of age in athletes without disabilities [31]. Furthermore, Ferrari [46] highlights that parents of disabled athletes tend to be more critical, having more difficulty managing their children’s emotional-motivational levels in the face of failure.

Another internal barrier where athletes with disabilities showed significantly higher scores than athletes without disabilities was *my current job does not allow me to study enough*. In this sense, it is important to note that the lack of professionalization that sport often has, especially in the case of sport for people with disabilities, means that student-athletes must look for a job that allows them to generate income with which to live [9, 31]. This complicates an already difficult situation, as student-athlete-workers must find compatibility in a triple career, dividing their time between studies, sport and work [9, 31]. The differences found in this barrier between disabled and non-disabled athletes could be due to the fact that while the percentage of student-athletes who worked was similar, the number of hours spent working was significantly higher for disabled student-athletes. This need especially among disabled athletes to have a high workload to generate income while trying to achieve or maintain elite status in their sport and not to fail academically could be a major stress factor for disabled student-athletes [31] who would perceive work as a necessity but at the same time as a distraction that takes time away from studying, an activity to which they end up devoting less time because it is the weakest link [9]. In addition to this, transport difficulties are accentuated for people with disabilities [28, 30, 32], which, added to the complicated time availability in the case of working, they see as an even greater barrier the fact that *the university is far from my training site* compared to non-disabled student-athlete-workers. Given these results, it is necessary to create a guaranteed system for student-athletes, especially those with disabilities, that offers them the security of being able to dedicate sufficient time to these tasks to be able to achieve success.

On the influence of gender on the perception of barriers, it was found that female student-athletes with disabilities perceived less support from the university (*I do not have enough university support*) than female student-athletes without disabilities. This could be due to the "double whammy" that disabled women usually suffer when they want to practice sports and become professionals in this field, as they are marginalized both because of their gender and their disability [47–50], in addition to having less structural and social [50], and financial support [51] in the pursuit of their dual career success, as well as negative experiences with male coaches who inappropriately addressed their gender and/or disability [52]. This could lead to a higher drop-out rate from sport or education among women [48], and therefore fewer women combine higher education and sports in adulthood. The results of the present research are in line with those found in previous studies which found that despite the fact that a large majority of higher educational institutions report addressing equal opportunities for students with disabilities in their universities [23], such initiatives do not seem to be making a deep impact, as students with disabilities report little support during post-secondary education institutions [8].

Another notable finding of the present research was that student-athletes with disabilities who had competed at the national level showed higher scores on the barrier *I have to take care of my family* than student-athletes without disabilities at the same competitive level. In this regard, the study conducted by Nair and Wade [53] concerning the life goals of people with disabilities, highlighted that the most rated one was family. This is evident in a growing number of studies documenting how people with disabilities engage in multifaceted caregiving as a significant part of their daily lives [54]. More recently, time use studies indicate that people with work disabilities or functional limitations [55] and people receiving Social Security Disability Insurance (SSDI) [54] spend non-negligible amounts of time each day on caregiving, with care for others sometimes being greater relative to parenting due to the fatigue associated with physical disability [56, 57], and with greater difficulties in accessing support than parents without disabilities [57].

On the other hand, among those student-athletes who had competed at the highest level, i.e. OG or PG, athletes with a disability showed higher scores on the external barrier *student schedules are not flexible*. Currently, despite the fact that 92% of university institutions address student disability [58], this does not seem to be reflected as students with disabilities report little support during post-secondary education [8, 25], a fact that may be aggravated when trying to combine studies with sport at the highest level, as high-level sport is undergoing a process of professionalization that requires athletes to dedicate a large part of their time to training and competition [59]. In this regard, students with disabilities highlight the lack of knowledge of lecturers about the theoretical-methodological basis, legal frameworks and policies for inclusion of people with disabilities, which in turn generates a feeling of inability and frustration [24], as well as the lack of attitudes and willingness of academic staff to provide adaptations [60].

It is worth noting that this is the first study that analyses the difficulties of student-athletes with disabilities for achieving success in their dual careers because of the barriers inherent in having to combine these two activities, in addition to the difficulties they encounter as a result of having a disability. As a result of the above, this study is pioneering, in terms of identifying the barriers that may be hindering the inclusion of disabled student-athletes in the education system, as compared to their non-disabled counterparts. Some measures that could help to overcome these barriers would be, firstly, to improve the lack of institutional coordination and multidisciplinary work between the different actors involved, i.e. education, sport, student-athlete and family [28, 61], in order to increase the chances of a successful dual career without having to give up any of the areas [15]. More specifically, from an educational perspective, it would be necessary to facilitate personalized educational programs for dual career students based on their specific needs [34], encouraging listening to disabled students and their organizations [62] and facilitating the flexibility of studies to be able to adapt them to each specific case. In particular, measures such as offering online, streaming or deferred classes, including additional exam sessions and attendance waivers, or giving athletes the possibility to train at the university’s own facilities would greatly facilitate the elimination of barriers related to travel and distance between centers for disabled student-athletes. Finally, some student-athletes also expressed the need to be supported through financial measures (scholarships), which could make it easier for some of them not to have to work outside their practice as professional athletes [9, 33, 62].

However, this research is not free of limitations. Firstly, the sample was chosen for convenience, which may not represent the generality of disabled and non-disabled student-athletes. Furthermore, the subjects analyzed were very heterogeneous in their socio-demographic characteristics (age and gender) and belonged to different sports and educational centers, including different levels, which entails completely different adaptations for the student-athletes, so these aspects should be considered in future research. Another limitation is that due to the heterogeneity of the sample and the lack of information on the adaptations carried out by the different study centers of origin for the success of the dual career, it was not possible to analyze the existence of any model that was perceived as more effective than another by the student athletes. Finally, another limitation of this research in relation to the sample of athletes with disabilities is that the results could be influenced by the type of disability of the athlete. It is also due to the accumulated wear and tear of many years of dual careers. Therefore, it is important for future research to collect information on these aspects at different universities and to analyze the student-athletes’ perception of these dimensions, as well as to find out whether the athletes’ opinions may be influenced by the type of disability they have or how many years they have been combining being a student with playing top-level sport.

In conclusion, student-athletes with disabilities scored the barriers to success in dual careers higher than non-disabled student-athletes, especially in terms of the distances between home, university and training, the difficulty in combining academic and sports performance with family care, and the feeling of being unable to find the right balance between study and training time. In addition, when a third activity is added to this dual career, such as work, they dedicate more hours to it, which makes them perceive that the job does not allow them to study enough. This is even though they find it more difficult to study and compete at a high level, which does not prevent them from perceiving to a greater extent, that sporting performance interferes with their studies. Therefore, it is necessary to take measures to ensure the inclusion of these student-athletes in the education system, so that the faced barriers are not the reason for them to abandon their dual career.
